# 2,3-Dimeth­oxy-5,12-tetra­cenequinone

**DOI:** 10.1107/S1600536809001147

**Published:** 2009-01-17

**Authors:** Chitoshi Kitamura, Naoki Akamatsu, Akio Yoneda, Takeshi Kawase

**Affiliations:** a Department of Materials Science and Chemistry, Graduate School of Engineering, University of Hyogo, 2167 Shosha, Himeji, Hyogo 671-2280, Japan

## Abstract

The mol­ecule of the title compound, C_20_H_14_O_4_, is approximately planar [maximum deviation 0.168 (2) Å]. The two meth­oxy groups are slightly twisted relative to the plane of the 5,12-tetra­cenequinone system, with twist angles of 3.3 (3) and 5.6 (2)°. All O atoms are involved in intermolecular C—H⋯O inter­actions and the mol­ecules are arranged into slipped face-to-face stacks along the *b* axis *via* π–π inter­actions with an inter­planar distance of 3.407 (2) Å.

## Related literature

For general background, see: Kitamura *et al.* (2008 ▶). For the synthetic procedures, see: McOmie & Perry (1973 ▶); Vets *et al.* (2004 ▶). For another synthetic method leading to the title compound, see: Reichwagen *et al.* (2005 ▶).
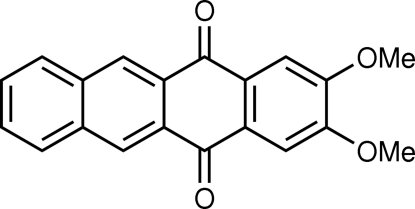


## Experimental

### 

#### Crystal data

C_20_H_14_O_4_
*M* *_r_* = 318.31
Monoclinic, 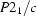

*a* = 8.290 (3) Å
*b* = 6.9781 (19) Å
*c* = 25.779 (8) Å
β = 97.883 (1)°
*V* = 1477.2 (8) Å^3^
*Z* = 4
Mo *K*α radiation
μ = 0.1 mm^−1^
*T* = 223 K
0.5 × 0.1 × 0.05 mm

#### Data collection

Rigaku Mercury CCD area-detector diffractometer
Absorption correction: numerical (*NUMABS*; Higashi, 1999 ▶) *T*_min_ = 0.988, *T*_max_ = 0.997
11405 measured reflections
3370 independent reflections
2773 reflections with *I* > 2σ(*I*)
*R*_int_ = 0.033

#### Refinement

*R*[*F* ^2^ > 2σ(*F* ^2^)] = 0.050
*wR*(*F* ^2^) = 0.147
*S* = 1.12
3370 reflections
219 parameters
H-atom parameters constrained
Δρ_max_ = 0.28 e Å^−3^
Δρ_min_ = −0.18 e Å^−3^

### 

Data collection: *CrystalClear* (Rigaku, 2001 ▶); cell refinement: *CrystalClear*; data reduction: *CrystalClear*; program(s) used to solve structure: *SIR2004* (Burla *et al.*, 2005 ▶); program(s) used to refine structure: *SHELXL97* (Sheldrick, 2008 ▶); molecular graphics: *ORTEP-3 for Windows* (Farrugia, 1997 ▶) and *Mercury* (Macrae *et al.*, 2006 ▶); software used to prepare material for publication: *WinGX* (Farrugia, 1999 ▶).

## Supplementary Material

Crystal structure: contains datablocks global, I. DOI: 10.1107/S1600536809001147/gk2182sup1.cif

Structure factors: contains datablocks I. DOI: 10.1107/S1600536809001147/gk2182Isup2.hkl

Additional supplementary materials:  crystallographic information; 3D view; checkCIF report

## Figures and Tables

**Table 1:** Hydrogen-bond geometry (Å, °)

*D*—H⋯*A*	*D*—H	H⋯*A*	*D*⋯*A*	*D*—H⋯*A*
C8—H8⋯O3^i^	0.94	2.30	3.210 (2)	162
C15—H15⋯O4^ii^	0.94	2.60	3.383 (2)	141
C20—H20*B*⋯O1^iii^	0.97	2.55	3.486 (2)	162
C20—H20*B*⋯O2^iii^	0.97	2.48	3.206 (2)	131

Symmetry codes: (i) 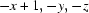; (ii) 
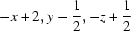
; (iii) 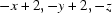.

**Fig. 1.**
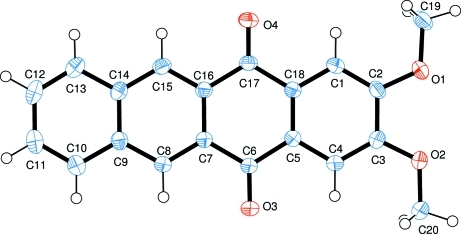
The molecular structure of the title compound showing 50% probability displacement ellipsoids for non-H atoms.

**Fig. 2.**
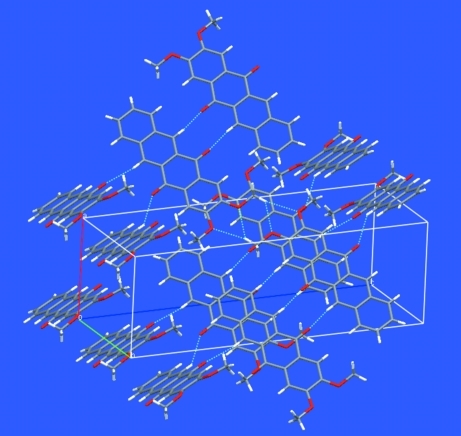
The packing diagram of the title compound. C-H···O interactions are shown with dashed lines.

## supplementary crystallographic information

### Comment

Although the title compound (I) was already synthesized (Reichwagen *et al.*, 2005), the X-ray structre was not reported. We prepared 2,3-dimethoxytetracene from 8,9-dimethoxy-5,12-tetracenequinone (McOmie & Perry, 1973), and attemped to perform the X-ray analysis of crystals made by recrystallization from a hot DMF solution under air and light. The analysis revealed that the molecule was not as expected 2,3-dimethoxytetracene but the title compound. Quinones have a weak dipole moment along the molecular long axis and are expected to take a antiparallel arrangement with respect to one another. The latter propensity may lead to the formation of face-to-face π-overlap along the stacking direction (Kitamura *et al.*, 2008).

The molecular structure is shown in Fig. 1. The molecule is approximately planar. The displacements of atoms O1, O2, O3, O4, C19, and C20 relative to the plane of the tetracene framework are -0.025 (1), -0.022 (1), -0.092 (1), 0.029 (1), -0.168 (2), and -0.113 (2) Å, respectively. The torsion angles of the two methoxy groups are -5.6 (2)° for C1—C2—O1—C19 and 3.3 (3)° for C4—C3—O2—C20, displaying that the C_methyl_—O bonds are directed along the molecular short axis.

In the crystal structure, the molecules are linked through intermolecular C—H···O hydrogen bonds between the methoxy groups as well as between the tetracene groups (Table 1, Fig. 2). Interestingly, along the stacking direction, not antiparallel but just slipped π-π stacking can be found. The interplanar distance is 3.407 (2) Å. The dipole moment of (I) was calculated by MO calculations (B3LYP/6–31G*), which afforded an estimation of 0.01 debye. Thus, (I) is a non-polar molecule. Therefore, it seems reasonably to conclude that the electrostatic property can determine either an antiparallel or a non-antiparallel arrangement.

### Experimental

8,9-Dimethoxy-5,12-tetracenequinone was prepared according to the method described by McOmie & Perry (1973). Transformation of tetracenequinone into tetracene was performed using two successive LiAlH_4_ reductions by Vets *et al.* (2004). To a suspension of LiAlH_4_ (224 mg, 5.9 mmol) in dry THF (15 ml), 8,9-dimethoxy-5,12-tetracenequinone (479 mg, 1.5 mmol) was added under nitrogen. The mixture was refluxed for 30 min, cooled to room temperature, and 6*M* HCl (7 ml) was added under cooling with ice. The residue was filtered, and washed with water, MeOH, and Et_2_O. After drying, a yellow solid was isolated. The solid was added into a suspension of LiAlH_4_ (235 mg, 6.2 mmol) in dry THF (15 ml). The mixture was again refluxed for 30 min, cooled to room temperature, and 6*M* HCl (7 ml) was added under cooling with ice. The product was filtered, and washed with water, MeOH, and Et_2_O. After drying, 2,3-dimethoxytetracene was obtained (287 mg, 66%) as a yellow solid. Heating the tetracene in DMF under air and light, and then cooling the solution to room temperature resulted in deposition of brown crystals suitable for X-ray analysis.

### Refinement

All H atoms were positioned geometrically and refined using a riding model approximation with C—H = 0.94Å and *U*_iso_(H) = 1.2*U*_eq_(C) for aromatic C—H, and C—H = 0.97Å and *U*_iso_(H) = 1.5*U*_eq_(C) for CH_3_.

### Crystal data

**Table:**

C_20_H_14_O_4_	*F*(000) = 664
*M**_r_* = 318.31	*D*_x_ = 1.431 Mg m^−^^3^
Monoclinic, *P*2_1_/*c*	Mo *K*α radiation, λ = 0.71073 Å
Hall symbol: -P 2ybc	Cell parameters from 4021 reflections
*a* = 8.290 (3) Å	θ = 3.0–27.5°
*b* = 6.9781 (19) Å	µ = 0.1 mm^−^^1^
*c* = 25.779 (8) Å	*T* = 223 K
β = 97.883 (1)°	Prism, brown
*V* = 1477.2 (8) Å^3^	0.5 × 0.1 × 0.05 mm
*Z* = 4	

### Data collection

**Table:**

Rigaku Mercury CCD area-detector diffractometer	3370 independent reflections
Radiation source: rotating-anode X-ray tube	2773 reflections with *I* > 2σ(*I*)
graphite	*R*_int_ = 0.033
Detector resolution: 14.7059 pixels mm^-1^	θ_max_ = 27.5°, θ_min_ = 3.0°
φ and ω scans	*h* = −10→10
Absorption correction: numerical (*NUMABS*; Higashi, 1999)	*k* = −9→5
*T*_min_ = 0.988, *T*_max_ = 0.997	*l* = −33→25
11405 measured reflections	

### Refinement

**Table:**

Refinement on *F*^2^	0 restraints
Least-squares matrix: full	H-atom parameters constrained
*R*[*F*^2^ > 2σ(*F*^2^)] = 0.050	*w* = 1/[σ^2^(*F*_o_^2^) + (0.0725*P*)^2^ + 0.2183*P*] where *P* = (*F*_o_^2^ + 2*F*_c_^2^)/3
*wR*(*F*^2^) = 0.147	(Δ/σ)_max_ < 0.001
*S* = 1.12	Δρ_max_ = 0.28 e Å^−^^3^
3370 reflections	Δρ_min_ = −0.18 e Å^−^^3^
219 parameters	

### Special details

**Table:**

Geometry. All e.s.d.'s (except the e.s.d. in the dihedral angle between two l.s. planes) are estimated using the full covariance matrix. The cell e.s.d.'s are taken into account individually in the estimation of e.s.d.'s in distances, angles and torsion angles; correlations between e.s.d.'s in cell parameters are only used when they are defined by crystal symmetry. An approximate (isotropic) treatment of cell e.s.d.'s is used for estimating e.s.d.'s involving l.s. planes.
Refinement. Refinement of *F*^2^ against ALL reflections. The weighted *R*-factor *wR* and goodness of fit *S* are based on *F*^2^, conventional *R*-factors *R* are based on *F*, with *F* set to zero for negative *F*^2^. The threshold expression of *F*^2^ > σ(*F*^2^) is used only for calculating *R*-factors(gt) *etc*. and is not relevant to the choice of reflections for refinement. *R*-factors based on *F*^2^ are statistically about twice as large as those based on *F*, and *R*- factors based on ALL data will be even larger.

### Fractional atomic coordinates and isotropic or equivalent isotropic displacement parameters (Å^2^)

**Table:**

	*x*	*y*	*z*	*U*_iso_*/*U*_eq_	
C1	1.00036 (17)	0.53181 (18)	0.14389 (6)	0.0275 (3)	
H1	1.062	0.5387	0.1773	0.033*	
C2	1.01106 (17)	0.67689 (19)	0.10804 (6)	0.0274 (3)	
C3	0.91825 (18)	0.66693 (19)	0.05767 (6)	0.0287 (3)	
C4	0.81764 (18)	0.51175 (19)	0.04488 (5)	0.0289 (3)	
H4	0.7562	0.5042	0.0115	0.035*	
C5	0.80647 (17)	0.36535 (18)	0.08134 (5)	0.0259 (3)	
C6	0.69108 (19)	0.20757 (19)	0.06595 (5)	0.0291 (3)	
C7	0.67888 (17)	0.05220 (18)	0.10459 (5)	0.0255 (3)	
C8	0.57670 (18)	−0.09998 (19)	0.09083 (5)	0.0282 (3)	
H8	0.5149	−0.103	0.0574	0.034*	
C9	0.56328 (18)	−0.25179 (18)	0.12612 (6)	0.0268 (3)	
C10	0.46090 (19)	−0.4123 (2)	0.11233 (6)	0.0341 (3)	
H10	0.4002	−0.4192	0.0788	0.041*	
C11	0.4501 (2)	−0.5567 (2)	0.14754 (7)	0.0384 (4)	
H11	0.3814	−0.6619	0.1381	0.046*	
C12	0.5408 (2)	−0.5489 (2)	0.19769 (6)	0.0386 (4)	
H12	0.5326	−0.6492	0.2215	0.046*	
C13	0.6411 (2)	−0.3969 (2)	0.21228 (6)	0.0344 (4)	
H13	0.7013	−0.3936	0.2459	0.041*	
C14	0.65452 (17)	−0.24356 (18)	0.17669 (6)	0.0273 (3)	
C15	0.75879 (18)	−0.08529 (19)	0.19038 (5)	0.0280 (3)	
H15	0.8193	−0.0794	0.224	0.034*	
C16	0.77246 (16)	0.05988 (18)	0.15515 (5)	0.0241 (3)	
C17	0.88638 (17)	0.22181 (18)	0.16997 (5)	0.0260 (3)	
C18	0.89773 (17)	0.37413 (18)	0.13063 (5)	0.0247 (3)	
C19	1.1882 (2)	0.8638 (2)	0.16855 (6)	0.0360 (4)	
H19A	1.1096	0.8696	0.1932	0.054*	
H19B	1.2491	0.9828	0.1699	0.054*	
H19C	1.2624	0.7579	0.1777	0.054*	
C20	0.8334 (2)	0.8166 (2)	−0.02447 (6)	0.0398 (4)	
H20A	0.8583	0.7051	−0.0444	0.06*	
H20B	0.8538	0.932	−0.0435	0.06*	
H20C	0.7199	0.8127	−0.0192	0.06*	
O1	1.10487 (14)	0.83614 (14)	0.11692 (4)	0.0362 (3)	
O2	0.93439 (14)	0.81632 (14)	0.02532 (4)	0.0378 (3)	
O3	0.60656 (17)	0.20730 (16)	0.02332 (4)	0.0491 (4)	
O4	0.96858 (15)	0.22784 (14)	0.21323 (4)	0.0401 (3)	

### Atomic displacement parameters (Å^2^)

**Table:**

	*U*^11^	*U*^22^	*U*^33^	*U*^12^	*U*^13^	*U*^23^
C1	0.0273 (7)	0.0287 (7)	0.0248 (7)	−0.0028 (5)	−0.0018 (5)	−0.0021 (5)
C2	0.0268 (7)	0.0268 (6)	0.0283 (7)	−0.0069 (5)	0.0027 (6)	−0.0035 (5)
C3	0.0311 (8)	0.0287 (7)	0.0263 (7)	−0.0044 (5)	0.0042 (6)	0.0040 (5)
C4	0.0322 (8)	0.0306 (7)	0.0223 (7)	−0.0061 (6)	−0.0021 (6)	0.0023 (5)
C5	0.0276 (7)	0.0244 (6)	0.0247 (7)	−0.0025 (5)	0.0004 (6)	0.0008 (5)
C6	0.0352 (8)	0.0265 (6)	0.0234 (7)	−0.0061 (6)	−0.0035 (6)	0.0017 (5)
C7	0.0291 (7)	0.0238 (6)	0.0229 (7)	−0.0019 (5)	0.0007 (6)	0.0005 (5)
C8	0.0317 (8)	0.0269 (6)	0.0244 (7)	−0.0037 (5)	−0.0022 (6)	0.0007 (5)
C9	0.0275 (7)	0.0237 (6)	0.0295 (8)	0.0003 (5)	0.0047 (6)	−0.0002 (5)
C10	0.0355 (8)	0.0301 (7)	0.0364 (8)	−0.0049 (6)	0.0033 (7)	−0.0009 (6)
C11	0.0388 (9)	0.0271 (7)	0.0505 (10)	−0.0064 (6)	0.0107 (7)	0.0001 (6)
C12	0.0453 (9)	0.0287 (7)	0.0442 (10)	0.0007 (6)	0.0143 (8)	0.0117 (6)
C13	0.0396 (9)	0.0323 (7)	0.0315 (8)	0.0033 (6)	0.0057 (7)	0.0074 (6)
C14	0.0293 (8)	0.0244 (6)	0.0286 (8)	0.0033 (5)	0.0059 (6)	0.0034 (5)
C15	0.0317 (8)	0.0279 (6)	0.0231 (7)	0.0023 (5)	−0.0004 (6)	0.0021 (5)
C16	0.0267 (7)	0.0217 (6)	0.0232 (7)	0.0017 (5)	0.0008 (5)	−0.0005 (5)
C17	0.0289 (7)	0.0253 (6)	0.0222 (7)	0.0010 (5)	−0.0026 (6)	−0.0009 (5)
C18	0.0261 (7)	0.0239 (6)	0.0234 (7)	−0.0006 (5)	0.0009 (5)	−0.0009 (5)
C19	0.0372 (9)	0.0353 (7)	0.0345 (8)	−0.0117 (6)	0.0013 (7)	−0.0091 (6)
C20	0.0465 (10)	0.0386 (8)	0.0318 (8)	−0.0136 (7)	−0.0034 (7)	0.0106 (6)
O1	0.0425 (7)	0.0324 (5)	0.0320 (6)	−0.0160 (4)	−0.0008 (5)	−0.0007 (4)
O2	0.0448 (7)	0.0347 (5)	0.0314 (6)	−0.0160 (5)	−0.0034 (5)	0.0090 (4)
O3	0.0679 (9)	0.0436 (6)	0.0288 (6)	−0.0257 (6)	−0.0190 (6)	0.0104 (5)
O4	0.0514 (7)	0.0337 (5)	0.0294 (6)	−0.0079 (5)	−0.0149 (5)	0.0029 (4)

### Geometric parameters (Å, °)

**Table:**

C1—C2	1.3818 (19)	C11—C12	1.405 (2)
C1—C18	1.4041 (18)	C11—H11	0.94
C1—H1	0.94	C12—C13	1.368 (2)
C2—O1	1.3577 (16)	C12—H12	0.94
C2—C3	1.417 (2)	C13—C14	1.4237 (19)
C3—O2	1.3530 (16)	C13—H13	0.94
C3—C4	1.3790 (19)	C14—C15	1.4170 (19)
C4—C5	1.3998 (19)	C15—C16	1.3757 (19)
C4—H4	0.94	C15—H15	0.94
C5—C18	1.3881 (19)	C16—C17	1.4885 (18)
C5—C6	1.4766 (18)	C17—O4	1.2254 (16)
C6—O3	1.2198 (17)	C17—C18	1.4810 (18)
C6—C7	1.4851 (18)	C19—O1	1.4262 (18)
C7—C8	1.3743 (18)	C19—H19A	0.97
C7—C16	1.4233 (18)	C19—H19B	0.97
C8—C9	1.4107 (19)	C19—H19C	0.97
C8—H8	0.94	C20—O2	1.4331 (18)
C9—C14	1.416 (2)	C20—H20A	0.97
C9—C10	1.4206 (19)	C20—H20B	0.97
C10—C11	1.368 (2)	C20—H20C	0.97
C10—H10	0.94		
			
C2—C1—C18	120.24 (12)	C13—C12—H12	119.6
C2—C1—H1	119.9	C11—C12—H12	119.6
C18—C1—H1	119.9	C12—C13—C14	120.25 (14)
O1—C2—C1	125.09 (13)	C12—C13—H13	119.9
O1—C2—C3	114.90 (12)	C14—C13—H13	119.9
C1—C2—C3	120.01 (12)	C9—C14—C15	119.45 (12)
O2—C3—C4	124.42 (13)	C9—C14—C13	118.93 (13)
O2—C3—C2	116.09 (12)	C15—C14—C13	121.61 (13)
C4—C3—C2	119.48 (12)	C16—C15—C14	120.81 (13)
C3—C4—C5	120.40 (13)	C16—C15—H15	119.6
C3—C4—H4	119.8	C14—C15—H15	119.6
C5—C4—H4	119.8	C15—C16—C7	119.53 (12)
C18—C5—C4	120.29 (12)	C15—C16—C17	119.76 (12)
C18—C5—C6	122.03 (12)	C7—C16—C17	120.70 (12)
C4—C5—C6	117.64 (12)	O4—C17—C18	121.27 (12)
O3—C6—C5	120.94 (12)	O4—C17—C16	120.91 (12)
O3—C6—C7	121.31 (12)	C18—C17—C16	117.82 (12)
C5—C6—C7	117.73 (12)	C5—C18—C1	119.56 (12)
C8—C7—C16	120.32 (12)	C5—C18—C17	121.15 (12)
C8—C7—C6	119.16 (12)	C1—C18—C17	119.27 (12)
C16—C7—C6	120.53 (12)	O1—C19—H19A	109.5
C7—C8—C9	120.93 (13)	O1—C19—H19B	109.5
C7—C8—H8	119.5	H19A—C19—H19B	109.5
C9—C8—H8	119.5	O1—C19—H19C	109.5
C8—C9—C14	118.95 (12)	H19A—C19—H19C	109.5
C8—C9—C10	121.86 (13)	H19B—C19—H19C	109.5
C14—C9—C10	119.19 (13)	O2—C20—H20A	109.5
C11—C10—C9	120.42 (14)	O2—C20—H20B	109.5
C11—C10—H10	119.8	H20A—C20—H20B	109.5
C9—C10—H10	119.8	O2—C20—H20C	109.5
C10—C11—C12	120.41 (14)	H20A—C20—H20C	109.5
C10—C11—H11	119.8	H20B—C20—H20C	109.5
C12—C11—H11	119.8	C2—O1—C19	117.45 (11)
C13—C12—C11	120.79 (13)	C3—O2—C20	117.27 (11)
			
C18—C1—C2—O1	179.31 (13)	C10—C9—C14—C13	0.1 (2)
C18—C1—C2—C3	0.0 (2)	C12—C13—C14—C9	−0.3 (2)
O1—C2—C3—O2	−0.27 (19)	C12—C13—C14—C15	−179.20 (14)
C1—C2—C3—O2	179.11 (13)	C9—C14—C15—C16	−0.1 (2)
O1—C2—C3—C4	−179.36 (13)	C13—C14—C15—C16	178.82 (13)
C1—C2—C3—C4	0.0 (2)	C14—C15—C16—C7	0.9 (2)
O2—C3—C4—C5	−178.75 (14)	C14—C15—C16—C17	−178.27 (12)
C2—C3—C4—C5	0.3 (2)	C8—C7—C16—C15	−0.6 (2)
C3—C4—C5—C18	−0.6 (2)	C6—C7—C16—C15	179.76 (13)
C3—C4—C5—C6	177.37 (13)	C8—C7—C16—C17	178.55 (13)
C18—C5—C6—O3	176.55 (15)	C6—C7—C16—C17	−1.1 (2)
C4—C5—C6—O3	−1.3 (2)	C15—C16—C17—O4	0.0 (2)
C18—C5—C6—C7	−2.0 (2)	C7—C16—C17—O4	−179.14 (13)
C4—C5—C6—C7	−179.93 (13)	C15—C16—C17—C18	179.63 (12)
O3—C6—C7—C8	3.6 (2)	C7—C16—C17—C18	0.5 (2)
C5—C6—C7—C8	−177.82 (13)	C4—C5—C18—C1	0.6 (2)
O3—C6—C7—C16	−176.79 (14)	C6—C5—C18—C1	−177.27 (13)
C5—C6—C7—C16	1.8 (2)	C4—C5—C18—C17	179.34 (13)
C16—C7—C8—C9	−0.5 (2)	C6—C5—C18—C17	1.5 (2)
C6—C7—C8—C9	179.12 (13)	C2—C1—C18—C5	−0.3 (2)
C7—C8—C9—C14	1.3 (2)	C2—C1—C18—C17	−179.09 (13)
C7—C8—C9—C10	−178.72 (13)	O4—C17—C18—C5	178.92 (13)
C8—C9—C10—C11	−179.71 (14)	C16—C17—C18—C5	−0.7 (2)
C14—C9—C10—C11	0.3 (2)	O4—C17—C18—C1	−2.3 (2)
C9—C10—C11—C12	−0.4 (2)	C16—C17—C18—C1	178.11 (12)
C10—C11—C12—C13	0.2 (2)	C1—C2—O1—C19	−5.6 (2)
C11—C12—C13—C14	0.1 (2)	C3—C2—O1—C19	173.78 (13)
C8—C9—C14—C15	−1.0 (2)	C4—C3—O2—C20	3.3 (2)
C10—C9—C14—C15	179.02 (13)	C2—C3—O2—C20	−175.73 (13)
C8—C9—C14—C13	−179.95 (13)		

### Hydrogen-bond geometry (Å, °)

**Table:**

*D*—H···*A*	*D*—H	H···*A*	*D*···*A*	*D*—H···*A*
C8—H8···O3^i^	0.94	2.30	3.210 (2)	162
C15—H15···O4^ii^	0.94	2.60	3.383 (2)	141
C20—H20B···O1^iii^	0.97	2.55	3.486 (2)	162
C20—H20B···O2^iii^	0.97	2.48	3.206 (2)	131

Symmetry codes: (i) −*x*+1, −*y*, −*z*; (ii) −*x*+2, *y*−1/2, −*z*+1/2; (iii) −*x*+2, −*y*+2, −*z*.
